# Acute disseminated encephalomyelitis presenting as fever of unknown origin: case report

**DOI:** 10.1186/1471-2431-11-103

**Published:** 2011-11-10

**Authors:** Margherita Di Costanzo, Maria Erminia Camarca, Maria Giovanna Colella, Giuseppe Buttaro, Andrea Elefante, Roberto Berni Canani

**Affiliations:** 1 Department of Pediatrics, University of Naples "Federico II", Naples, Italy; 2 Pediatric Unit, Formia Hospital, Formia, Italy; 3 Department of Neurological Sciences, University of Naples "Federico II", Naples, Italy

## Abstract

**Background:**

Fever of unknown origin (FUO) can be defined as a body temperature higher than 38.3°C on several occasions over more than 3 weeks, the diagnosis of which remains uncertain after 1 week of evaluation. Acute disseminated encephalomyelitis (ADEM) is an inflammatory demyelinating disease of the central nervous system with a wide range of clinical manifestations. The highest incidence of ADEM is observed during childhood and it usually occurs following a viral or bacterial infection or, more rarely, following a vaccination, or without a preceding cause.

**Case presentation:**

Here, we describe an atypical case of ADEM that initially manifested as several weeks of FUO in a fifteen years old boy.

**Conclusions:**

This case report suggests a new possible syndromic association between ADEM and FUO, which should be considered in the clinical examination of patients with FUO, especially in the presence of also modest neurologic or neuropsychiatric symptoms.

## Background

Fever of unknown origin (FUO) can be defined as a body temperature higher than 38.3°C on several occasions over more than 3 weeks, the diagnosis of which remains uncertain after 1 week of evaluation [1]. Evaluation of FUO in children is complex, because of a wide range of possible etiologies. The most common causes of FUO in childhood are viral infections, while in older patients frequent causes are inflammatory illnesses (rheumatic diseases, vasculitides, polymyalgia rheumatic, sarcoidosis), infections and neoplasms [1,2]. Acute disseminated encephalomyelitis (ADEM) is uncommon inflammatory demyelinating disease of the central nervous system (CNS) with a wide range of clinical manifestations. The highest incidence of ADEM is observed during childhood and it usually occurs following a viral or bacterial infection or, more rarely, following a vaccination, or without a preceding cause [3]. Onset of the disorder is sudden. According to the classic definition, ADEM is a monophasic disease, but it can also present a relapsing course, being described as "recurrent" if the affected districts are always the same or "multiphasic" if there is dissemination in space and time of the lesions [4]. Irritability and lethargy are common first signs of ADEM. Fever and headache are reported about in half of patients. Fever in ADEM is frequently associated with the development of neurologic symptoms, which can occur after hours or weeks from the onset of illness. The most common neurologic symptoms are visual field deficits; language disturbances; mental status abnormalities ranging from irritability and lethargy to coma; psychiatric changes which include depression, personality changes and psychosis. Meningeal signs are reported in children with severe disease. Weakness, which may be hemiparetic or generalized and symmetric, is more commonly detected than sensory defects. Other reported symptoms are cranial nerve palsies, generalized or focal seizures and ataxia [5-8]. Here we describe an atypical case of ADEM presenting as FUO.

## Case presentation

**Figure 1:**
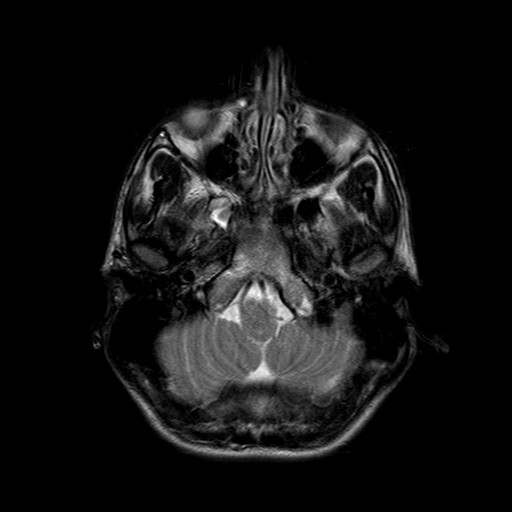
**Transverse T2-weighted MRI brain image shows small high-signal lesions on the lateral right side of the pontine tegmentum**.

The patient was an Italian boy of fifteen years old who was admitted to the pediatric department of the University of Naples "Federico II" for persistent fever from 25 days with inconstant headache, asthenia and a state of anxiety. He was in a poor state of health. The clinical examination didn't reveal any sign of localization of fever. Familial history was unremarkable except for his sister who had used drugs in the past and was suffering from hepatitis C virus (HCV) infection. Personal history revealed only allergic rhinitis with positive skin prick test. The past medical history revealed that he had a motorcycle accident seven months before. On that occasion a CT of skull was negative. Six months before hospital admission he presented flu like syndrome. A few months before he showed a state of anxiety characterized by tachycardia and agitation. For these symptoms a neurologist recommended a drug therapy (levosulpiride, ademetionine and hypothalamic phospholipid liposomes). One week prior to admission at our hospital, he was admitted at the pediatric unit of Formia hospital for high-spiking fever, which was poor responsive to paracetamol, and inconstant headache. During the previous admission, a definitive documentation of fever and exclusion of factitious fever were obtained. A total body CT scanning was performed in order to exclude consequences of the previous motorcycle accident. The following laboratory studies, which were carried out on several occasions, resulted within the normal range: complete blood count (CBC); peripheral blood smear; inflammatory indexes (erythrocyte sedimentation rate (ESR), C-reactive protein (CRP), serum protein electrophoresis (SPEP), assay of immunoglobulins) (Table 1); serum chemistry; urine and blood culture; throat and urethral swab; serology for viral hepatitis, human immunodeficiency virus (HIV), cytomegalovirus (CMV), Epstein-Barr virus (EBV), herpes simplex viruses (HSV), rubella and bartonella infection, brucellosis, chlamydial diseases, typhoid and paratyphoid A and B fever, rickettsiosis, syphilis and toxoplasmosis; Mantoux test and rapid test for Malaria; immunologic screening (antinuclear antibodies, antimitochondrial antibodies, rheumatologic factor and C3-C4); thyroid hormones, cortisol, ACTH, aldosterone, angiotensin-converting enzyme; blood lead and toxicological investigations for benzodiazepines, opiates, methadone, cocaine, cannabis, amphetamines. The patient also underwent the following exams: chest and skull radiography, abdominal ultrasonography, total body CT scanning and color flow Doppler echocardiography, but these imaging studies failed to disclose the diagnosis. Appropriate consultations were indicated based on patient history, including the following: infectious disease specialist, hematologist and neurologist. Hematologist asked for a bone marrow aspirate, which resulted negative for the research of leukemia and other myeloproliferative disorders. This detailed workup was helpful to exclude the most common causes of FUO: factitious fever, infectious diseases, neoplasms, immunodeficiencies, autoimmune diseases, vasculitides, endocrine disorders, drug fever and inflammatory bowel diseases. Despite negative exams, he still had fever and was in a poor state of health. The clinical examination did not show any sign of localization of fever. His headache and panic state got worse, so an immediate ophthalmologic visit was performed. The exam of *fundus oculi *showed: fade borders of optical disc, raised on retinal surface (left eye > right eye). Normal macula. Increased vascular tortuosity. A cranial magnetic resonance (MRI) was urgently ordered. The MRI showed some areas of hyperintensity in T2-weighted and Fluid-attenuated Inversion Recovery (FLAIR) images. These areas were in several regions of brain. They were typical of an inflammatory autoimmune disease, like ADEM (Figure 1). The next day the patient underwent an EEG, which showed suffering of temporal right areas of brain. A lumbar puncture with cerebrospinal fluid (CSF) analysis revealed elevated protein content (92 mg/dl), normal glucose content (5.2 mmol/L, compared to a random blood glucose level 7.5 mmol/L) and leucocytosis, predominantly lymphocytosis (100 lymphocytes cell/mm^3^). The opening pressure was normal (17 cm CSF). CSF oligoclonal bands of IgG were negative. Their presence was more often associated with Multiple Sclerosis (MS), the most important alternative diagnosis to ADEM. CSF culture and an extensive microbiologic workup for bacterial and viral infection of CNS have been helpful in distinguishing ADEM from various infectious forms of meningoencephalitis. Acute infectious encephalitis may be caused by a wide range of viruses but the most important is herpes simplex encephalitis (HSE) because of its severity, especially if untreated, and its good response to specific treatment with acyclovir. Analysis of the CSF for HSV DNA using the Polymerase Chain Reaction (PCR) has been a significant advance in the diagnosis of HSE as this test has a very high sensitivity and specificity especially with appropriate sample timing [9]. In our patient serology for HSV and analysis of CSF for HSV DNA resulted negative, thus the diagnosis of HSE was ruled out. CNS vasculitides may also result in syndromes resembling ADEM, but negative serum markers of inflammation, immunologic screening and CSF analysis didn't favor this diagnostic hypothesis. Moreover neuroimaging showed demyelization areas, typical of an autoimmunitary inflammatory disease like ADEM, so we immediately started therapy with high doses of glucocorticoids e.v. The patient received 1 g/die of methylprednisolone for 5 days, and then he continued the therapy with progressive decreased doses of methylprednisolone by oral way [4,8]. Patient conditions quickly improved. Fever disappeared and headache decreased. He still had panic and anxiety, so he started a therapy with dipotassium clorazepate (5 mg/die), with improvement of symptoms. After 6 days of therapy, the patient repeated ophthalmologic visit; *fundus oculi *still showed mild signs of intracranial hypertension. After 13 days of therapy patient repeated cranial MRI, which showed complete resolution of the lesions. The patient was discharged in a good state of health, with a domiciliary therapy. He stopped glucocorticoids therapy after 1 month. He also underwent a last ophthalmologic visit and the *fundus oculi *was normal. After three and six months, at follow up examination no neurologic symptoms were found. At the follow up we re-evaluated the diagnosis of ADEM, because many patients initially diagnosed with ADEM develop clinically definite MS. The final diagnosis of ADEM was only established because there was no evidence of a second clinical and neuroradiologic episode of CNS demyelination.

**Table 1:** Laboratory data: complete and differential blood count, inflammatory indexes (erythrocyte sedimentation rate (ESR), C-reactive protein (CRP), serum protein electrophoresis (SPEP), assay of immunoglobulins).

White blood cells (× 10^3^/μL)	8.81
Red blood cells (× 10^6^/μL)	5.02

Platelets (× 10^3^/μL)	256

Hemoglobin (g/dl)	12.9

Hematocrit (%)	38.3

Neutrophils (%)	71.9

Lymphocytes (%)	19.7

Monocytes (%)	7.5

Eosinophils (%)	0.6

Basophils (%)	0.3

ESR (mm/h)	10

CRP (mg/L)	0.03

Total protein (g/dl)	6.6

IgG (mg/dl)	1112

IgA (mg/dl)	309

IgM (mg/dl)	74

Albumin (g/dL)	64.4

Alpha 1 (g/dL)	2.7

Alpha 2 (g/dL)	10.1

Beta (g/dL)	9.0

Gamma (g/dL)	13.8

A/G	1.81

## Conclusions

Acute disseminated encephalomyelitis (ADEM) is an immune-mediated inflammatory disorder of the CNS characterized by a widespread demyelization that predominantly involves the white matter of the brain and spinal cord. The condition is usually precipitated by a viral infection or vaccination [10] and is more frequent in children. Today the exact incidence of ADEM is little known. In one study of children with ADEM living in San Diego County, California, the incidence was estimated to be at least 0.4/100.000/year [11], but in the last decade ADEM has been increasingly diagnosed as more magnetic resonance imaging studies are performed on patients with acute encephalopathy. Based on its immune-mediated etiology, ADEM is commonly treated with high-dose steroids. Factors influencing treatment efficacy and possible alternative options for steroid resistant cases remain unclear. In a portion of patients who fail to respond to steroid therapy, intravenous immunoglobulin has been used with some benefit. Unsolved issues regard clinical predictors to best select therapy for groups of patients. Plasmapheresis and cytostatic drugs are alternative treatment options in patients who do not respond to steroid and/or to intravenous immunoglobulins [8]. An acute encephalopathy with multifocal neurologic signs and deficits is usually the first clinical manifestation, but ADEM has a wide range of presenting features. We described an atypical case of ADEM that initially manifested as several weeks of FUO. A successful diagnosis of the underlying disease required an intensive and rational diagnostic evaluation of the wide spectrum of possible etiologies of FUO. This case report suggests a new possible syndromic association between ADEM and FUO, which should be considered in the clinical examination of patients with FUO, especially in the presence of also modest neurologic or neuropsychiatric symptoms.

## Consent

Written informed consent was obtained from the parents of the patient for publication of this case report. A copy of the written consent is available for review by the Editor-in-Chief of this journal.

## Competing interests

The authors declare that they have no competing interests.

## Authors' contributions

All authors contributed equally to this work, read and approved the final manuscript.

## Pre-publication history

The pre-publication history for this paper can be accessed here:

http://www.biomedcentral.com/1471-2431/11/103/prepub
